# Function Characterization of Endogenous Plasmids in *Cronobacter sakazakii* and Identification of *p*-Coumaric Acid as Plasmid-Curing Agent

**DOI:** 10.3389/fmicb.2021.687243

**Published:** 2021-06-25

**Authors:** Xuemeng Ji, Ping Lu, Yaozhong Hu, Juan Xue, Jing Wu, Bowei Zhang, Yan Zhang, Lu Dong, Huan Lv, Shuo Wang

**Affiliations:** ^1^Tianjin Key Laboratory of Food Science and Health, School of Medicine, Nankai University, Tianjin, China; ^2^Institute of Radiation Medicine, Chinese Academy of Medical Sciences and Peking Union Medical Collage, Tianjin, China; ^3^Institute of Infection and Immunity, Taihe Hospital, Hubei University of Medicine, Shiyan, China

**Keywords:** plasmid curing, virulence, antibiotic resistance, *Cronobacter sakazakii*, *p*-coumaric acid

## Abstract

Virulence traits and antibiotic resistance are frequently provided by genes located on plasmids. However, experimental verification of the functions of these genes is often lacking due to a lack of related experimental technology. In the present study, an integrated suicide vector was used to efficiently and specifically delete a bacterial endogenous plasmid in *Cronobacter sakazakii*. The pESA3 plasmid was removed from *C. sakazakii* BAA-894, and we confirmed that this plasmid contributes to the invasion and virulence of this strain. In addition, the pGW1 plasmid was expunged from *C. sakazakii* GZcsf-1, and we confirmed that this plasmid confers multidrug resistance. We further screened plasmid-curing agents and found that *p*-coumaric acid had a remarkable effect on the curing of pESA3 and pGW1 at sub-inhibitory concentrations. Our study investigated the contribution of endogenous plasmids pESA3 and pGW1 by constructing plasmid-cured strains using suicide vectors and suggested that *p*-coumaric acid can be a safe and effective plasmid-curing agent for *C. sakazakii*.

## Introduction

Previously developed strategies for the treatment of systemic infections caused by pathogenic bacteria still rely on the application of various antibiotics. However, the frequent and extensive utilization of antibiotics has dramatically increased the possibility of severe infections related to bacteria with multidrug resistance, posing a more aggressive threat to public health and necessitating the development of novel antimicrobials and bacterial vaccines. Moreover, prospective research on the underlying mechanism of multidrug resistance could facilitate the modification of currently employed antibiotics and the development of novel reagents. To date, multidrug resistance has been observed in a variety of pathogenic bacteria, including *Neisseria gonorrhoeae* (Yuan et al., 2019; Chen et al., 2020; Seong et al., 2020), *Pseudomonas aeruginosa* (Horcajada et al., 2019; Kumarage et al., 2019; Tada et al., 2019), and *Acinetobacter baumannii* (da Silva et al., 2018; Salehi et al., 2018).

Related studies have unveiled the inseparable correlation between bacterial antibiotic resistance and plasmids, which could be verified by the frequent distribution of virulence determinants and antibiotic resistance genes in plasmids (Stratev and Odeyemi, 2016; Kopotsa et al., 2019). Importantly, the abuse of antibiotics promoted the spread of antibiotic-resistant plasmids that have the potential to increase the occurrence of virulence or drug resistance genes, posing an increased threat to society (Fraise, 2002; Zhang et al., 2011; Mangat et al., 2017).

Bacterial plasmids can significantly vary in size from 2 to 2,000 kbp (Canteros, 1990). Large plasmids can potentially harbor more transcriptomic information and perform relatively important functions that contribute to host survival.

Recently, *Cronobacter sakazakii*, an emerging pathogenic bacterium belonging to the family Enterobacteriaceae, has attracted a great deal of attention due to its multidrug resistance and ability to cause disease with a high mortality rate (Parra-Flores et al., 2018; Odeyemi and Abdullah Sani, 2019). *C. sakazakii* is a foodborne pathogen associated with life-threatening sepsis (Hassan and Naser, 2018), meningitis (Chaves et al., 2018; Zeng et al., 2018), and necrotizing enterocolitis (Fan et al., 2019) in premature and full-term infants. *C. sakazakii* has been widely isolated and identified from powder infant formula (PIF) (Li et al., 2019), fruit powders, vegetables, tea leaves, herbs, cereals, and spices (Shi et al., 2016).

The pathogenicity and multidrug resistance of *C. sakazakii* are believed to be associated with two large plasmids that it harbors, pESA3 and pGW1, with sizes of 134 and 340 kbp, respectively. A variety of virulence-related genes have been identified in pESA3 (Aly et al., 2019), and pGW1 has been reported to carry a number of drug resistance genes (Zeng et al., 2018).

The highly conserved replicon of RepFIB in pESA3 and its frequent identification in almost 97% of *Cronobacter* isolates has been previously described (Singh et al., 2017). pESA3 encodes two iron uptake systems (*lucABCD*/*lutA* and *EitCBAD*) that may facilitate host survival and pathogenesis. In 2018, one multidrug-resistant strain of *C. sakazakii*, GZcsf-1, was reported to be responsible for a case of meningitis in a neonate in China (Zeng et al., 2018). Two internalized plasmids, namely, pGW1 (340 kbp) and pGW2 (135 kbp), were identified, with 17 predicted drug resistance genes encoded by pGW1 and a high similarity observed for pGW2 to pESA3 (Zeng et al., 2018). Moreover, an *mcr-1*-harboring plasmid identified in *C. sakazakii* was confirmed to be correlated with colistin resistance (Liu et al., 2017; Nang et al., 2019), which is widely used as an antibiotic of last-resort against bacterial infections. Considering the ubiquitous presence of *C. sakazakii* in nature, as well as its excellent capacity for plasmid internalization, it may be associated with an increased risk of potential infections and an increased threat to public health, especially in infants.

Given the frequent observation of plasmid-mediated bacterial drug resistance, functional characterization of these plasmids is still needed. Currently, such functional studies primarily rely on comparative genomic analysis without further experimental verification, which may be attributed to the difficulty of transforming plasmids into *Escherichia coli* to further study their functions, especially large plasmids (Tu et al., 2016; Chung et al., 2017). Moreover, the common presence of multiple replicons in large plasmids impedes the elucidation of their function through replicon incompatibility-mediated plasmid curing (Medaney et al., 2016; Douarre et al., 2020).

In this study, we used an integrated suicide vector to efficiently and specifically delete an endogenous plasmid of choice in *C. sakazakii* and analyzed the contributions of endogenous plasmids pESA3 and pGW1. The plasmid-cured strains were used to screen plasmid-curing agents and showed that *p*-coumaric acid had a remarkable effect on the plasmid curing, suggesting that *p*-coumaric acid can be a safe and effective plasmid-curing agent for *C. sakazakii*.

## Results

### Strategy of Plasmid Curing in *C. sakazakii*

**FIGURE 1:**
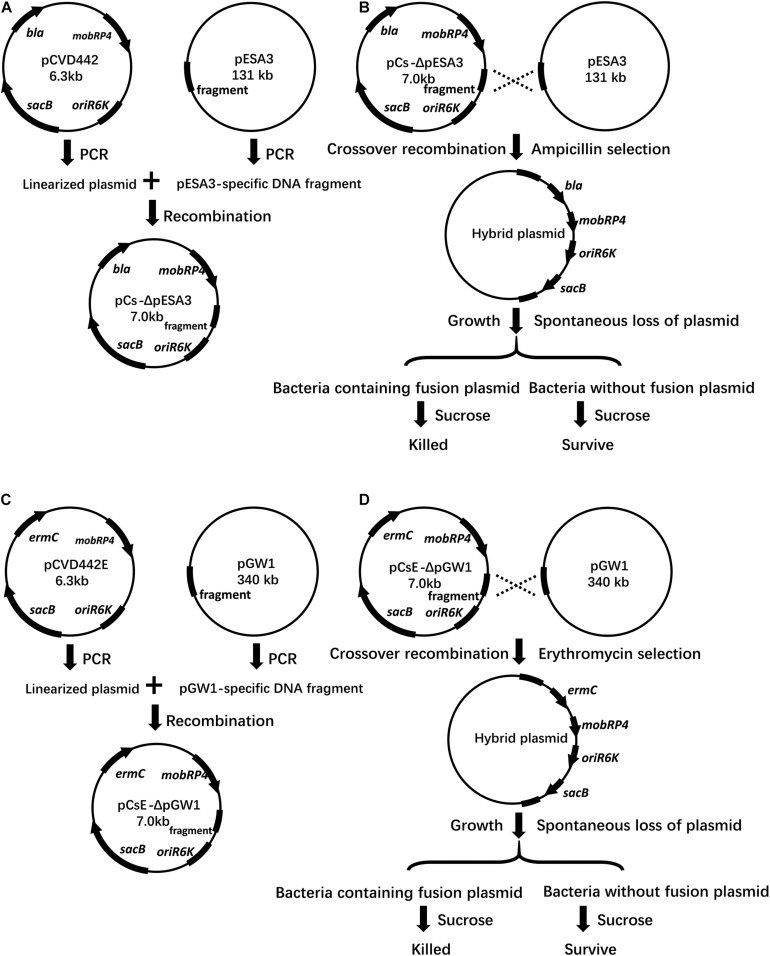
Schematic representation of vectors, crossover recombination, plasmid hybrid, and sucrose selection. **(A)** The vector pCs-ΔpESA3 is shown with the pESA3-specific hybrid fragment and gene orientation of the ampicillin resistance (*bla*), *pir* replication initiator (oriR6K), sucrose sensitivity marker (*sacB*) and broad-host-range mobilizable region (mobRP4). pCVD442 was linearized by PCR and ligated with a pESA3-specific DNA fragment, resulting in the plasmid pCs-ΔpESA3. **(B)** The plasmid pCs-ΔpESA3 was extracted from S17–1 lambda pir *E. coli* and transformed into *C. sakazakii* BAA-894. The integration of pCs-ΔpESA3 into the endogenous plasmid pESA3 of *C. sakazakii* BAA-894 by crossover recombination resulted in the strain *C. sakazakii* BAA-894 containing the hybrid plasmid. Growth of the plasmid crossover recombinants allowed for the spontaneous loss of the hybrid plasmid in a small fraction of the bacteria. Exposure to sucrose resulted in selective killing of the bacteria containing the hybrid plasmid that retained the sucrose sensitivity marker *sacB*, and the surviving bacteria cured of the plasmid were identified by testing for ampicillin susceptibility and via plasmid-specific PCR. **(C)** The vector pCs-ΔpGW1 was similar to pCsE-ΔpESA3, but *ermC* was used instead of *bla* due to the existence of the cephalosporin resistance gene *bla*_DHA__–__1_ in pESA3, and the fragment was amplified from pGW1. **(D)** The procedure was the same as that described in **(B)**, except the endogenous plasmid to be deleted was pGW1, and the integrated suicide plasmid was pCs-ΔpGW1. **(B,D)** The hybrid plasmid was used for plasmid complementation in the plasmid-cured strain.

**FIGURE 2:**
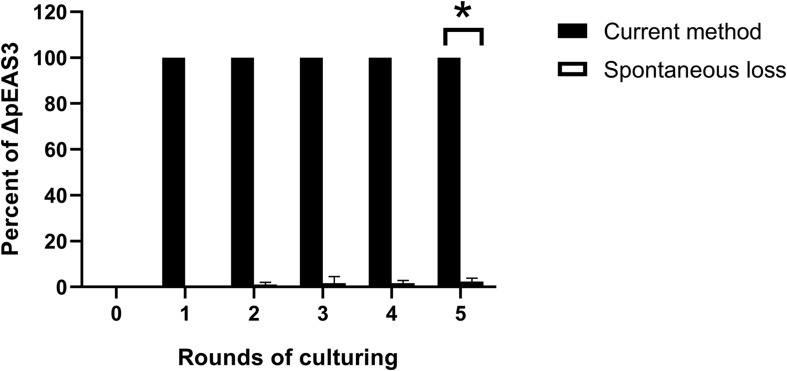
Percentage of pESA3 plasmid-cured isolates after continuous culturing. The method described in the present study represents a strategy for plasmid curing in *C. sakazakii*, and the spontaneous loss of an endogenous plasmid represents the traditional molecular method based on plasmid incompatibility using another plasmid with the same replicon. The loss of the plasmid was verified by plasmid-specific PCR using primers listed in Table 3. At the beginning of the experiment, 10^5^ CFU/mL bacteria harboring the integrated plasmid pCs-ΔpESA3 (Figure 1B) and bacteria harboring the incompatible plasmid pUC57-RepFIB were prepared and subsequently continuously cultured in LB containing sucrose and ampicillin, respectively. The cultures were shaken overnight at 37°C and then inoculated into 20 mL of fresh sterile LB medium at a 1:100 dilution the next day. After each round of culturing, bacterial samples were serially diluted and plated on agar to obtain signal colonies. Finally, 100 single colonies were selected, and their genotypes were determined by PCR using plasmid-specific primers. Each data point represents the average and standard deviation of three biological repeats. Student’s t-test; *P < 0.05.

A strategy based on suicide vector pCVD442 was developed for plasmid curing in the current study. Generally, the plasmid-specific fragment of the suicide vector was taken from an endogenous plasmid (Figure 1), integration of the hybrid plasmid could then be specifically selected using the antibiotic corresponding to the resistance marker on the suicide vector. Then, the suicide plasmid was spontaneously removed in culture without antibiotic stress, leaving a suspension of two types of bacteria with or without the hybrid plasmid. Finally, based on the sensitivity to sucrose, the bacteria with the hybrid plasmid were eliminated by sucrose supplementation. The loss of the plasmid was verified by plasmid-specific PCR. 58 out of randomly selected 60 colonies growing on LB plates containing sucrose in pESA3 and pGW1 curing assays were verified to be plasmid curing strains, indicating a high frequency of loss of the plasmid. One hundred single colonies were selected in the sucrose selection step and verified to be plasmid-cured isolates. Compared to the traditional molecular method based on plasmid incompatibility, where 92% (92/100) of *C. sakazakii* transformant colonies retained pESA3 after 5 rounds of culturing in the presence of ampicillin, the method developed in the present study resulted in 100% (100/100) of transformant colonies being verified as pESA3-cured isolates (Figure 2). These results clearly showed that this method is efficient for expunging plasmids in *C. sakazakii*.

**TABLE 1:** Antimicrobial drug susceptibility profiles.

Antibiotics	MIC, mg/L antimicrobial susceptibility*	
	*C. Sakazakii* GZcsf-1	*C. Sakazakii* GZcsf-1 ΔpGW1
Ampicillin	32/R	1/S
Ceftriaxone	≥64/R	≤0.13/S
Cefazolin	32/R	2/S
Chloramphenicol	≥32/R	4/S
Azithromycin	≥ 32/R	8/S
Gentamicin	≥32/R	≤0.13/S
Trimethoprim	≥128/R	1/S
Aztreonam	≥32/R	0.13/S
Tetracycline	≥32/R	1/S
Streptomycin	≥512/R	≥256/R
Spectinomycin	≥512/R	≥512/R

*The interpretation of the minimum inhibitory concentration (MIC) on bacterial resistance (sensitive, S; intermediate, I; and resistant, R) was performed using the Enterobacteriaceae data from the Clinical and Laboratory Standards Institute (CLSI).

**TABLE 2:** Bacterial strains and plasmids used in this study.

Strains, plasmids	Description	References, sources
**Cronobacter sakazakii**		
*C. sakazakii* BAA-894	International reference strain	ATCC
*C. sakazakii* BAA-894 ΔpESA3	pESA3 curing variant	This study
*C. sakazakii* GZcsf-1	Clinical strain	Zeng et al., 2018
*C. sakazakii* GZcsf-1 ΔpGW1	pGW1 curing variant	This study
**Escherichia coli**		
S17-1 lambda pir	Strain constructed that harbors the lambda pir gene	de Lorenzo et al., 1993
**Plasmids**		
pUC57-*ermC*	pUC57 vector harboring the Erm^§^ gene	Ji et al., 2019
pCVD442	Suicide plasmid	Donnenberg and Kaper, 1991
pCs-ΔpESA3	pESA3 integrated plasmid that originated from pCVD442	This study
pCVD442E	Erm^®^ plasmid that originated from pCVD442	This study
pCsE-ΔpGW1	pGW1 integrated plasmid that originated from pCVD442E	This study
pUC57-RepFIB	pUC57 vector harboring the RepFIB replicon cloned in AatII site using RepFIB-F-AatII and RepFIB-R-AatII primer pair	This study

**TABLE 3:** Primers used in this study.

Primer	Sequence (5′–3′)
pCVD442-F	GGCTGTCAGACCAAGTTTACTCATATATACT TTAGATTG
pCVD442-R	GCAGATACTCTTCCTTTTTCAATATTATTGAAGCATTTA TCAGGGTTATTG
ΔpESA3-F	GAAAAAGGAAGAGTATCTGCGGTACGGTACGGCC ATACTGTTCG
ΔpESA3-R	GCGATTAACCCATCTAAACGTCTCCACTAAA AAATCGTCATC
RepFIB-F-AatII	GCGACGTCTGAGCAAACATCCACTGTGG
RepFIB-R-AatII	GCGACGTCATAACGCATCAGTTGAAAAC
Check1-pESA3-F	GTCAACGGCACGATGGATCT
Check1-pESA3-R	CAGCGCCGATCGCCTGGCGC
Check2-pESA3-F	GAGCGGCAGTGTTGCCTGGC
Check2-pESA3-R	TCCAGCGTTGCGCTTTTTCA
*ermC*-F	GCTAGCCTTGACAATTAATCATCGGCTCGTATA ATGTCTAG
*ermC*-R	GTAAACTTGGTCTGACAGCCCTTAACTTACTTATTA AATAATTTATAGC
pCVD442E-F1	GGCTGTCAGACCAAGTTTACTCATATATAC TTTAGATTG
pCVD442E-R1	GCAGATACTCTTCCTTTTTCAATATTATTGAAGCATTTA TCAGGGTTATTG
ΔpGW1-F	GAAAAAGGAAGAGTATCTGCGGTTTACCGATCA GCGTTACC
ΔpGW1-R	TGTTAGCAGGCAGTTCCAGGGCCTATGTCG CCTTTATTCC
Check1-pGW1-F	CCGATCAGCGTTACCGGTGC
Check1-pGW1-R	TGGACGTTCTGCGTTTTATC
Check2-pGW1-F	ATTTGATCGGGTTTTATCGT
Check2-pGW1-R	TCTTGTGAAAGCCAGATTCA

### pESA3 Contributes to Invasion and Virulence

**FIGURE 3:**
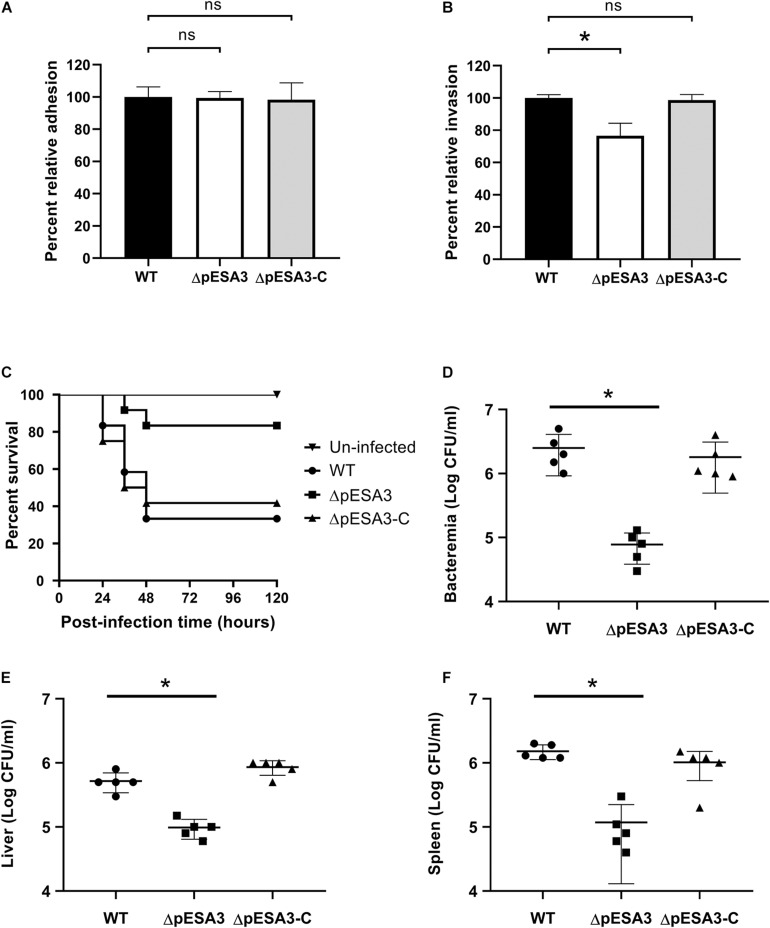
The endogenous plasmid pESA3 contributes to the virulence of *Cronobacter sakazakii* BAA-894. Adhesion **(A)** and invasion **(B)** of Caco-2 cells by WT, ΔpESA3 and ΔpESA3 complementation (ΔpESA3*-*C) *C. sakazakii* strains (*n* = 3). **(C)** Survival curves of rat pups 120 h after oral infection. **(D–F)** Colony-forming unit (CFU) counts at 24 h postinfection from the blood, liver, and spleen of orally infected rats. Blood samples were collected from facial veins, liver and spleen, homogenized and serially diluted. The diluents were plated on LB agar for colony enumeration. The data are presented as the means and standard deviation of five biological repeats (Student’s *t*-test; ns, no significant difference, *P < 0.05).

Many of the genes distributed on the pESA3 plasmid are predicted to be involved in the adhesion and infection of the host pathogens (Aly et al., 2019). To verify the role of pESA3 in pathogenicity, the *C. sakazakii* strain BAA-894 encapsulated with or without pESA3 was used to assess the contribution of pESA3 to the adhesion, invasion and virulence of the host. In addition, the hybrid plasmid was used for complementation pESA3 in the pESA3-cured strain (Figures 1C,D). *In vitro* analysis indicated that pESA3-cured bacteria were significantly attenuated in their ability to invade Caco-2 cells than the wild-type strain (Figure 3B), but no difference was observed with respect to adhesion (Figure 3A). In addition, the results of an *in vivo* study revealed that *C. sakazakii* BAA-894 lacking pESA3 had notably lower lethality than wild-type bacteria after intragastric administration to 3-day-old rats (Figure 3C). The survival rate of rats infected by wild-type bacteria was 40% at 120 h postinfection, while 80% survival was observed for those infected with pESA3-cured bacteria. Furthermore, the organs of young rats exhibited a significantly lower distribution of pESA3-cured bacteria 24 h postinfection compared to those infected with the wild-type or complemented strains (Figures 3D–F).

### pGW1 Is Involved in Multidrug Resistance

*C. sakazakii* GZcsf-1 was initially isolated from the brain abscess fluid of an infant meningitis patient, and multidrug resistance to various antibiotics has been reported to be potentially linked with the endogenous plasmid pGW1 (Zeng et al., 2018). However, the dominant role of pGW1 in multidrug resistance remains obscure and needs to be investigated. Therefore, in the present study, the antibiotic sensitivity of wild-type *C. sakazakii* GZcsf-1 and *C. sakazakii* GZcsf-1 without pGW1 was determined (Table 1). The results of a minimum inhibitory concentration (MIC) analysis demonstrated the resistance of wild-type *C. sakazakii* GZcsf-1 to multiple antibiotics, namely, ampicillin, ceftriaxone, cefazolin, chloramphenicol, azithromycin, gentamicin, trimethoprim, aztreonam, and tetracycline, while the cured pGW1 variant was susceptible to all these antibiotics. In addition, both *C. sakazakii* GZcsf-1 strains harboring or lacking pGW1 exhibited resistance to spectinomycin and streptomycin. These results clearly demonstrated the association of pGW1 with multidrug resistance in *C. sakazakii* GZcsf-1, although not against spectinomycin and streptomycin, which is probably dependent on genes present in the genome. Indeed, the presence of aminoglycoside adenyltransferase (*aadA1*), a gene that confers antibiotic resistance to spectinomycin and streptomycin, has been confirmed in the genome of *C. sakazakii* GZcsf-1 (Zeng et al., 2018), which could potentially explain the obtained results.

### Plasmid Curing of *C. sakazakii* With *p*-Coumaric Acid

**FIGURE 4:**
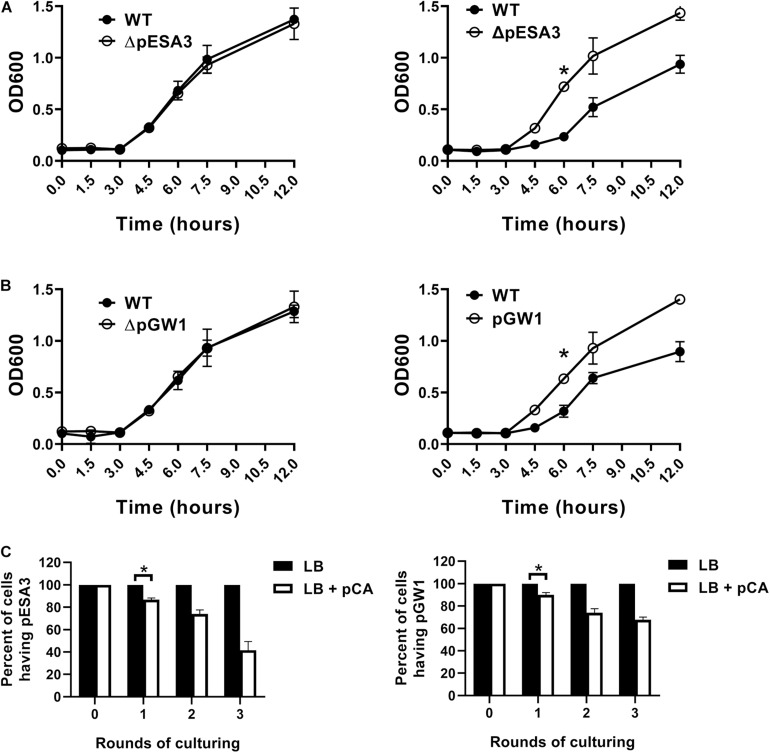
Plasmid-curing ability of *p*-coumaric acid in *Cronobacter sakazakii*. **(A)** Growth of wild-type *C. sakazakii* BAA-894 and its ΔpESA3 derivative in LB (left) and in LB containing 250 mg/L *p*-coumaric acid (right). **(B)** Growth of wild-type *C. sakazakii* GZcsf-1 and its ΔpGW1 derivative in LB (left) and in LB containing 250 mg/L *p*-coumaric acid (right). **(C)** Percent of plasmid-cured isolates of each culture round in LB or in LB containing 250 mg/L *p*-coumaric acid (pCA). Left: initial strain was wild-type *C. sakazakii* BAA-894, right: initial strain was wild-type *C. sakazakii* GZcsf-1. At the beginning of the experiment, a total of 10^5^ CFU of wild-type bacteria was added to 10 mL of LB or LB containing pCA. The cultures were shaken overnight at 37°C and then inoculated into 10 mL of fresh sterile LB medium or LB containing pCA at a 1:100 dilution the next day, respectively. After each round of culturing, bacterial samples were serially diluted and plated on agar to obtain signal colonies. Finally, 100 single colonies were selected, and their genotypes were determined by PCR using plasmid-specific primers. All data represent the average and standard deviation of three biological repeats (Student’s *t*-test; *P < 0.05).

It has been found that plant-derived chemicals have the effect of plasmid elimination (39). Considering that polyphenols are naturally existing substances in food and are relatively safe, we next selected plasmid elimination drugs from polyphenols. The plasmid knockout strains obtained in our project were used to screen plasmid elimination drugs by comparing the growth rates of plasmid knockout strains and wild-type strains at sub-inhibitory concentrations of drugs. The minimal inhibitory concentration (MIC) of 12 polyphenols, including daidzin, trans-chalcone, apigenin, quercetin, flavanone, polydatin, trihydroxyflavone, caffeic acid, 5,7-Dihydroxyflavone, *p*-coumaric acid, isoferulic acid and phenethyl cinnamate, were determined using broth dilution methods. However, the MICs of 13 of these polyphenols were above 1,000 mg/L, and only *p*-coumaric acid had a MIC of 500 mg/L for both *C. sakazakii* GZcsf-1 and *C. sakazakii* BAA-894, irrespective of bacterial antibiotic resistance behavior. Thus, the plasmid-curing effects of these polyphenols at 250 mg/L were tested by comparing the growth rates between plasmid knockout strains and wild-type strains. In LB, wild-type *C. sakazakii* BAA-894 showed a similar growth rate as *C. sakazakii* BAA-894 ΔpESA3, and wild-type *C. sakazakii* GZcsf-1 showed a similar growth rate as *C. sakazakii* GZcsf-1 ΔpGW1 (Figure 4A). However, in LB complemented with 250 mg/L *p*-coumaric acid, wild-type *C. sakazakii* BAA-894 showed a significant reduction in optical density at 600 nm (OD600) compared to that of the *C. sakazakii* BAA-894 ΔpESA3 strain (Figure 4B). Similar results were also observed in *C. sakazakii* GZcsf-1 and its pGW1 curing mutant, indicating an *in vitro* fitness cost of the endogenous plasmids imposed on *C. sakazakii* with the existence of *p*-coumaric acid. Subsequently, the plasmid curing effect of *p*-coumaric acid was directly examined by continuous culture of wild-type *C. sakazakii* in LB or LB with *p*-coumaric acid. We found that with the increase of culture cycles, the proportion of plasmid-eliminating bacteria of *C. sakazakii* in the medium supplemented with *p*-coumaric acid increased gradually, while no plasmid-cured bacteria were detected in the medium without *p*-coumaric acid (Figure 4C). These results revealed that *p*-coumaric acid exhibits plasmid-curing effect on *C. sakazakii* strains.

## Discussion

*C. sakazakii* is an opportunistic pathogen that is widely distributed in foods (Zeng et al., 2020), the gut (Chandrasekaran et al., 2018) and various environments, increasing its chance of coming into contact with a variety of antibiotics, which may account for the accumulation of drug resistance genes in this bacterium, especially multidrug resistance genes in plasmids or the genome. In particular, the ability of *C. sakazakii* to harbor plasmids larger than 300 kbp would further increase its propensity to acquire resistance (Zeng et al., 2018). The resistance plasmids harbored by *C. sakazakii* could potentially serve as reservoirs for and as the origin for resistance in other strains through conjugation, transduction and transformation. The investigated fragment in pGW1 from *C. sakazakii* is also conserved in plasmids from *S. enterica*, *S. flexneri*, *C. freundii*, and *K. pneumoniae*, which could explain the dissemination of drug resistance plasmids in different bacteria.

The conventional strategy used to study the function of plasmids primarily depends on the removal of endogenous plasmids or their exogenous transformation into *E. coli* for functionality analysis. The exogenous transformation of plasmids of interest can promote a better understanding of their relationship to resistance to specific antibiotics but cannot provide sufficient insight into their roles in bacterial pathogenicity. In addition, the lower transformation efficiency of large plasmids or their restricted replication in *E. coli* can further restrict the application of the traditional strategy used to evaluate plasmids. The typical approaches used to cure endogenous plasmids primarily include chemical-based methods and molecular biological methods. Common chemical methods depend on the use of a high concentration of SDS, EDTA or ethidium bromide to interfere with plasmid replication, which has certain disadvantages, including non-specific removal, increased tendency for genomic mutation, and high toxicity (Buckner et al., 2018). Alternatively, molecular biology methods based on plasmid incompatibility involve the construction of plasmids containing replicons to cure endogenous plasmids, which primarily rely on the accurate cloning of the replicon from endogenous plasmids (Buckner et al., 2018). However, this method is subject to the prediction of plasmid incompatibility, and the multiple replicons that are frequently present in endogenous plasmids can further restrict the applicability of the plasmid incompatibility-based method (Citterio et al., 2020). Another traditional approach uses transposons harboring the *sacB* or *rpsL* genes to cure various plasmids that were established decades ago. However, transposons have insertion sites, and the frequency of the occurrence of hybridization of the plasmid to be cured and transposon is low, resulting in a time-consuming colony screening process (Hynes et al., 1989; Stojiljković et al., 1991; Brom et al., 1992). A method based on the CRISPR/Cas9 system and homing endonuclease shows the capability of plasmid curing in certain species (Cao et al., 2017; Lauritsen et al., 2017; Tang et al., 2018; Wang et al., 2019). However, the off-target effect of the system leads to the concern over unwanted cleavages and mutations in chromosomal DNA. Furthermore, intracellular DNA ligase could repair the CRISPR/Cas9 system/endonuclease-induced DNA double-strand breaks, and an additional procedure to inhibit activity of DNA ligase has been proposed (Weber et al., 2015), casting a shadow over the application of this plasmid-curing method. Recently, suicide vectors were used in specific plasmid curing in *Agrobacterium* and *Salmonella* strains (Yamamoto et al., 2018; Yin et al., 2018), suggesting that this method has the advantages of plasmid specificity and convenience in plasmid elimination, but its broad-spectrum application needs more verification.

The current research described the integrated suicide vector pCVD442 to efficiently and specifically delete an endogenous plasmid of choice in *C. sakazakii* and analyzed the contribution of endogenous plasmids pESA3 and pGW1. The plasmid elimination method provided in the current study could facilitate the generation of plasmid knockout strains and could be applied for evaluation of selected plasmid elimination drugs by comparisons of their growth rates with those of wild-type strains under fixed pressure. Furthermore, the lower toxicity of the plasmid knockout strains could contribute to the development of attenuated vaccines after detailed investigation.

Plant-derived chemicals have many functions in the field of microorganisms, including antibacterial activity, bactericidal activity, regulation of quorum sensing and so on. The plasmid-cured strains obtained in our study were used to screen plasmid-curing agents from plant polyphenols and found that *p*-coumaric acid had a remarkable effect on the plasmid curing. Previous studies have found that *p*-coumaric acid can compete with ethidium bromide in binding to DNA (Lou et al., 2012), suggesting that *p*-coumaric acid can intercalate into the DNA base pairs. In fact, many DNA intercalating agents including ethidium bromide, such as methyl orange and acriflavine, have been found to eliminate plasmids from various strains (Buckner et al., 2018). Therefore, the plasmid-curing activity of *p*-coumaric acid in *C. sakazakii* may due to intercalation of *p*-coumaric into plasmid DNA, blocking the replication of plasmid. Previous studies have also found some polyphenols with plasmid elimination activity for specific bacteria. It seems that the plasmid elimination activity of plant polyphenols is not broad-spectrum, which may be due to the different tolerances of different bacteria to polyphenols. Our study found that *p*-coumaric acid inhibited the growth of *C. sakazakii* at high concentrations and had plasmid elimination effects at low concentrations. This is the first report on the antibacterial effect of *p*-coumaric acid on *C. sakazakii*. Considering the safety of *p*-coumaric acid derived from food, it may be a potential food additive for the inhibition of *C. sakazakii*. In addition, this is the first report in which *p*-coumaric acid can inhibit the plasmid-carrying capacity of *C. sakazakii* at sub-inhibitory concentrations. Most importantly, we found that the antibiotic resistance of *C. sakazakii* was not related to the elimination and growth inhibition of its plasmid by *p*-coumaric acid. Therefore, *p*-coumaric acid may solve the multiple drug resistance problem of *C. sakazakii*.

Overall, the current research analyzed the contributions of the endogenous plasmids pESA3 and pGW1 by constructing plasmid-cured strains using an integrated suicide vector. The plasmid-cured strains were used to screen plasmid-curing agents and showed that *p*-coumaric acid derived from plants had a remarkable effect on the plasmid curing, suggesting that *p*-coumaric acid can be a safe and effective plasmid-curing agent for *C. sakazakii*.

## Materials and Methods

### Strains and Plasmids

All strains and plasmids used in the present study are listed in Table 1, and primers are listed in Table 2. Bacteria were stored in LB medium (Solarbio, China) containing 15% glycerol (Solarbio, China) at –80°C. To initiate all experiments, strains were cultured overnight in LB. If necessary, antibiotics (ampicillin or kanamycin) were added at a final concentration of 100 μg/mL (Solarbio, China), and *E. coli* S17–1 lambda pir (Weidi, China) was used to prepare the pCVD442 suicide vector (Miaoling, China). The gene cloning and transformation of *C. sakazakii* were performed using standard techniques.

### Curing of Endogenous Plasmids

To remove pESA3 from the *C. sakazakii* strain BAA-894, the specific fragment amplified from the plasmid to be cured using the primer pair ΔpESA3-F and ΔpESA3-R was mixed with the linearized plasmid pCVD442 (Miaoling, China) using the primer pair pCVD442-F and pCVD442-R, and plasmid cyclization was achieved by homogenous recombination using a recombination kit according with protocols recommended by the manufacturer (Vazyme, China). The hybrid plasmid was selected by transformation into S17–1 lambda pir competent cells (Weidi, China) and selective medium. Single colonies growing on LB agar were selected. Subsequently, the plasmid was extracted from the prepared S17–1 lambda pir-Δplasmid, and *C. sakazakii* BAA-894 was transformed by electroporation as previously described (Kim and Loessner, 2008). A colony was cultured in LB overnight and then transferred to LB agar containing 20% sucrose. Strains harboring the integrated suicide plasmid will be killed by sucrose due to harboring *sacB* in the plasmid, while strains that lost the plasmid will survive. The genotype of colonies growing on sucrose LB agar was further verified by PCR and sequencing using dual pESA3 plasmid-specific primer pairs (see primer pairs, Table 3).

To cure pGW1 from the strain *C. sakazakii* GZcsf-1, the specific fragment that was amplified from the plasmid to be removed using the primer pair ΔpGW1-F and ΔpGW1-R was mixed with plasmid pCVD442 that was linearized using the primer pair pCVD442-F1 and pCVD442-R1 and the *ermC* fragment amplified from pUC57-*ermC* (Ji et al., 2019) using the primer pair *ermC*-F and *ermC*-R. The following procedure was identical as the protocols described for the removal of pESA3, but 300 mg/L erythromycin was used to select strains harboring the hybrid plasmid. The genotype of colonies growing on sucrose agar was further verified by PCR and sequencing using dual pGW1 plasmid-specific primer pairs (see primer pairs, Table 3).

### Generation of the Plasmid Complemented Strains

Hybrid plasmid was used for plasmid complementation in the corresponding plasmid-cured strain (Figures 1C,D). Specifically, the hybrid plasmid was extracted from S17–1 lambda pir-Δplasmid, and plasmid-cured *C. sakazakii* BAA-894 was transformed by electroporation as previously described (Kim and Loessner, 2008). The plasmid complemented strains were selected by plating the bacteria on LB agar containing corresponding antibiotics. The genotype of colonies growing on selective medium was further verified by PCR and sequencing using dual plasmid-specific primer pairs (see primer pairs, Table 3).

### Susceptibility Assays

The antimicrobial sensitivity of *C. sakazakii* strains was tested by the agar dilution method according to Performance Standards for Antimicrobial Disk Susceptibility Tests, 13th Edition^1^. All antibiotics were purchased from Beijing Solarbio Science and Technology Co., Ltd., China. All polyphenols were purchased from Shanghai yuanye Bio-Technology Co., Ltd., China. Briefly, bacteria cultured overnight were diluted in LB media to 10^7^ CFU/mL, and 10 μL of liquid was aliquoted on LB agar plates containing a two-fold dilution series of antimicrobials. The MICs were determined as the lowest concentration at which growth could not be observed when the plates were incubated at 37°C for 24–48 h.

### Adhesion Assay

Bacterial adhesion assays were performed as described previously (Choi et al., 2015). Briefly, Caco-2 cells were washed in PBS (Solarbio, China) and then resuspended in fresh RPMI 1640 medium. *C. sakazakii* was prepared and added to a Caco-2 cell monolayer at a multiplicity of infection (MOI) of 100. After incubating for 45 min, the cells were washed with PBS three times and lysed in 1% Triton X-100. Then, the suspension was serially diluted and plated on LB agar to enumerate the CFU.

### Invasion Assays

Bacterial invasion assays were performed as described previously (Choi et al., 2015). In short, Caco-2 cells were washed in PBS, and fresh RPMI 1640 media was added. Then, *C. sakazakii* cells were prepared and added to a Caco-2 cell monolayer at an MOI of 100. After incubating for 90 min, the cells were washed with PBS three times and then incubated with 100 μg/mL gentamycin for 1 h to kill extracellular bacteria. Subsequently, the wells were washed with PBS three times and lysed in 1% Triton X-100. Then, the suspension was serially diluted and plated on LB agar to enumerate the CFU.

### *In vivo* Rat Pup Virulence Assay

Bacterial cells were washed and resuspended in PBS (Solarbio, China), and a mixed inoculum of 5 × 10^9^ CFU of bacteria was orally administered to 3-days-old female Sprague-Dawley rat pups (4 rats/group). Where appropriate, chemicals were orally administered 1 h after bacterial administration. To analyze the colonization of bacteria in organs, after 24 h of infection, the bacterial load in the blood was determined by diluting facial vein blood and plating the diluents on LB agar. Then, the rats were sacrificed, and the liver and spleen were aseptically removed. The organs were homogenized in ice-cold PBS and serially diluted. The diluent was plated on LB agar to determine the bacterial loads.

### Statistical Analysis

Statistical significance was analyzed using the GraphPad Prism (version 8.4) with the unpaired *t*-test. The data are represented as the mean and standard deviation. A *P*-value of < 0.05 was considered to indicate a significant difference.

## Data Availability Statement

The original contributions presented in the study are included in the article/supplementary material, further inquiries can be directed to the corresponding author/s.

## Ethics Statement

The animal study was reviewed and approved by the Nankai University.

## Author Contributions

XJ and PL conceived, designed the research, conducted the experiments, and analyzed the data. YH and XJ contributed new reagents or analytical tools. JW, BZ, YZ, LD, and HL provided useful suggestions. SW systematically reviewed the literature and wrote the manuscript. All authors contributed to the editing of the manuscript.

## Conflict of Interest

The authors have filed a China patent application (No. 202010973714.6) based on the results reported in this manuscript.

## Footnotes

1 https://clsi.org/standards/products/microbiology/documents/

## References

[B1] Aly M. A. Domig K. J. Kneifel W. Reimhult E. (2019). Whole genome sequencing-based comparison of food isolates of *Cronobacter sakazakii*. *Front. Microbiol.* 10:1464. 10.3389/fmicb.2019.01464 31333604 PMC6615433

[B2] Brom S. De Los Santos A. G. Stepkowsky T. Flores M. Dávila G. Romero D. (1992). Different plasmids of *Rhizobium leguminosarum* bv. phaseoli are required for optimal symbiotic performance. *J. Bacteriol.* 174 5183–5189. 10.1128/jb.174.16.5183-5189.1992 1644746 PMC206350

[B3] Buckner M. M. C. Ciusa M. L. Piddock L. J. V. (2018). Strategies to combat antimicrobial resistance: anti-plasmid and plasmid curing. *FEMS Microbiol. Rev.* 42 781–804. 10.1093/femsre/fuy031 30085063 PMC6199537

[B4] Canteros B. I. (1990). *Diversity of Plasmids and Plasmid-Encoded Phenotypic Traits in Xanthomonas campestris pv. vesicatoria.* Ph.D thesis. Gainesville, FL: University of Florida.

[B5] Cao Q.-H. Shao H.-H. Qiu H. Li T. Zhang Y.-Z. Tan X.-M. (2017). Using the CRISPR/Cas9 system to eliminate native plasmids of Zymomonas mobilis ZM4. *Biosci. Biotechnol. Biochem.* 81 453–459. 10.1080/09168451.2016.1189312 27900888

[B6] Chandrasekaran S. Burnham C. D. Warner B. B. Tarr P. I. Wylie T. N. (2018). Carriage of *Cronobacter sakazakii* in the very preterm infant gut. *Clin. Infect. Dis.* 67 269–274. 10.1093/cid/ciy062 29394356 PMC6030953

[B7] Chaves C. E. V. Brandão M. L. L. Lacerda M. L. G. G. Rocha C. A. B. C. (2018). Fatal *Cronobacter sakazakii* sequence type 494 meningitis in a newborn, Brazil. *Emerg. Infect. Dis.* 24:1948. 10.3201/eid2410.180373 30226186 PMC6154150

[B8] Chen S.-C. Hu L.-H. Zhu X.-Y. Yin Y.-P. (2020). Gonococcal urethritis caused by a multidrug resistant *Neisseria gonorrhoeae* strain with high-level resistance to spectinomycin in China. *Emerg. Microbes Infect.* 9 517–519. 10.1080/22221751.2020.1732836 32116136 PMC7067170

[B9] Choi Y. Kim S. Hwang H. Kim K. P. Kang D. H. Ryu S. (2015). Plasmid-encoded MCP is involved in virulence, motility, and biofilm formation of *Cronobacter sakazakii* ATCC 29544. *Infect. Immun* 83 197–204. 10.1128/iai.02633-14 25332122 PMC4288869

[B10] Chung M. E. I Yeh H. Sung L. Y. Wu M. Y. Chao Y. P. I Ng S. (2017). Enhanced integration of large DNA into *E. coli* chromosome by CRISPR/Cas9. *Biotechnol. Bioeng.* 114 172–183. 10.1002/bit.26056 27454445

[B11] Citterio B. Andreoni F. Simoni S. Carloni E. Magnani M. Mangiaterra G. (2020). Plasmid replicon typing of antibiotic-resistant *Escherichia coli* from clams and marine sediments. *Front. Microbiol.* 11:1101. 10.3389/fmicb.2020.01101 32528456 PMC7266932

[B12] da Silva K. E. Maciel W. G. Croda J. Cayô R. Ramos A. C. de Sales R. O. (2018). A high mortality rate associated with multidrug-resistant *Acinetobacter baumannii* ST79 and ST25 carrying OXA-23 in a Brazilian intensive care unit. *PLoS One* 13:e0209367. 10.1371/journal.pone.0209367 30592758 PMC6310363

[B13] de Lorenzo V. Eltis L. Kessler B. Timmis K. N. (1993). Analysis of *Pseudomonas* gene products using lacIq/Ptrp-lac plasmids and transposons that confer conditional phenotypes. *Gene* 123 17–24. 10.1016/0378-1119(93)90533-9 8380783

[B14] Donnenberg M. S. Kaper J. B. (1991). Construction of an eae deletion mutant of enteropathogenic *Escherichia coli* by using a positive-selection suicide vector. *Infect. Immun.* 59 4310–4317. 10.1128/iai.59.12.4310-4317.1991 1937792 PMC259042

[B15] Douarre P.-E. Mallet L. Radomski N. Felten A. Mistou M.-Y. (2020). Analysis of COMPASS, a new comprehensive plasmid database revealed prevalence of multireplicon and extensive diversity of IncF plasmids. *Front. Microbiol.* 11:483. 10.3389/fmicb.2020.00483 32265894 PMC7105883

[B16] Fan H. Chen Z. Lin R. Liu Y. Wu X. Puthiyakunnon S. (2019). *Bacteroides* fragilis strain ZY-312 defense against *Cronobacter sakazakii*-induced necrotizing enterocolitis in vitro and in a neonatal rat model. *Msystems* 4 e00305–e00319. 31387931 10.1128/mSystems.00305-19 PMC6687943

[B17] Fraise A. P. (2002). Biocide abuse and antimicrobial resistance—a cause for concern? *J. Antimicrob. Chemother.* 49 11–12. 10.1093/jac/49.1.11 11751760

[B18] Hassan J. S. Naser W. E. (2018). Incidence of *Cronobacter sakazakii* in Iraqi Infants with neonatal sepsis. *Indian J. Public Health Res. Dev.* 9 908–913. 10.5958/0976-5506.2018.01256.1

[B19] Horcajada J. P. Montero M. Oliver A. Sorlí L. Luque S. ómez-Zorrilla S. G. (2019). Epidemiology and treatment of multidrug-resistant and extensively drug-resistant *Pseudomonas aeruginosa* infections. *Clin. Microbiol. Rev.* 32:e00031–19. 31462403 10.1128/CMR.00031-19 PMC6730496

[B20] Hynes M. F. Quandt J. O’Connell M. P. Pühler A. (1989). Direct selection for curing and deletion of Rhizobium plasmids using transposons carrying the *Bacillus subtilis* sacB gene. *Gene* 78 111–120. 10.1016/0378-1119(89)90319-3 2548927

[B21] Ji X. Lu P. van der Veen S. (2019). Development of a dual-antimicrobial counterselection method for markerless genetic engineering of bacterial genomes. *Appl. Microbiol. Biotechnol.* 103 1465–1474. 10.1007/s00253-018-9565-5 30607491

[B22] Kim K.-P. Loessner M. J. (2008). *Enterobacter sakazakii* invasion in human intestinal Caco-2 cells requires the host cell cytoskeleton and is enhanced by disruption of tight junction. *Infect. Immun.* 76 562–570. 10.1128/iai.00937-07 18070906 PMC2223463

[B23] Kopotsa K. Osei Sekyere J. Mbelle N. M. (2019). Plasmid evolution in carbapenemase-producing *Enterobacteriaceae*: a review. *Ann. N. Y. Acad. Sci.* 1457 61–91. 31469443 10.1111/nyas.14223

[B24] Kumarage J. Khonyongwa K. Khan A. Desai N. Hoffman P. Taori S. K. (2019). Transmission of multi-drug resistant *Pseudomonas aeruginosa* between two flexible ureteroscopes and an outbreak of urinary tract infection: the fragility of endoscope decontamination. *J. Hosp. Infect.* 102 89–94. 10.1016/j.jhin.2019.02.015 30802523

[B25] Lauritsen I. Porse A. Sommer M. O. A. Nørholm M. H. H. (2017). A versatile one-step CRISPR-Cas9 based approach to plasmid-curing. *Microb. Cell Fact.* 16:135. 28764701 10.1186/s12934-017-0748-z PMC5540278

[B26] Li C. Zeng H. Zhang J. He W. Ling N. Chen M. (2019). Prevalence, antibiotic susceptibility, and molecular characterization of *Cronobacter* spp. isolated from edible mushrooms in China. *Front. Microbiol.* 10:283. 10.3389/fmicb.2019.00283 30863374 PMC6399401

[B27] Liu B.-T. Song F.-J. Zou M. Hao Z.-H. Shan H. (2017). Emergence of colistin resistance gene mcr-1 in *Cronobacter sakazakii* producing NDM-9 and in *Escherichia coli* from the same animal. *Antimicrob. Agents Chemother.* 61:e01444–16. 27855074 10.1128/AAC.01444-16 PMC5278688

[B28] Lou Z. Wang H. Rao S. Sun J. Ma C. Li J. (2012). *p*-coumaric acid kills bacteria through dual damage mechanisms. *Food Control* 25 550–554. 10.1016/j.foodcont.2011.11.022

[B29] Mangat C. S. Bekal S. Irwin R. J. Mulvey M. R. (2017). A novel hybrid plasmid carrying multiple antimicrobial resistance and virulence genes in *Salmonella enterica* serovar Dublin. *Antimicrob. Agents Chemother.* 61 e02601–e02616. 28320711 10.1128/AAC.02601-16 PMC5444150

[B30] Medaney F. Ellis R. J. Raymond B. (2016). Ecological and genetic determinants of plasmid distribution in *Escherichia coli*. *Environ. Microbiol.* 18 4230–4239. 10.1111/1462-2920.13552 27696647

[B31] Nang S. C. Li J. Velkov T. (2019). The rise and spread of mcr plasmid-mediated polymyxin resistance. *Crit. Rev. Microbiol.* 45 131–161. 10.1080/1040841x.2018.1492902 31122100 PMC6625916

[B32] Odeyemi O. A. Abdullah Sani N. (2019). Antibiotic resistance, putative virulence factors and curli fimbrination among *Cronobacter* species. *Microb Pathog* 136:103665. 10.1016/j.micpath.2019.103665 31404630

[B33] Parra-Flores J. Aguirre J. Juneja V. Jackson E. E. Cruz-Córdova A. Silva-Sanchez J. (2018). Virulence and antibiotic resistance profiles of *Cronobacter sakazakii* and *Enterobacter* spp. involved in the diarrheic hemorrhagic outbreak in Mexico. *Front. Microbiol.* 9:2206. 10.3389/fmicb.2018.02206 30319560 PMC6171480

[B34] Salehi B. Goudarzi H. Nikmanesh B. Houri H. Alavi-Moghaddam M. Ghalavand Z. (2018). Emergence and characterization of nosocomial multidrug-resistant and extensively drug-resistant *Acinetobacter baumannii* isolates in Tehran, Iran. *J. Infect. Chemother.* 24 515–523. 10.1016/j.jiac.2018.02.009 29555392

[B35] Seong Y. J. Alhashimi M. Mayhoub A. Mohammad H. Seleem M. N. (2020). Repurposing fenamic acid drugs to combat multidrug-resistant *Neisseria gonorrhoeae*. *Antimicrob Agents Chemother.* 64 e02206–e02219. 32393483 10.1128/AAC.02206-19 PMC7318036

[B36] Shi C. Sun Y. Zhang X. Zheng Z. Yang M. Ben H. (2016). Antimicrobial effect of lipoic acid against *Cronobacter sakazakii*. *Food Control* 59 352–358. 10.1016/j.foodcont.2015.05.041

[B37] Singh N. Raghav M. Narula S. Tandon S. Goel G. (2017). Profiling of virulence determinants in *Cronobacter sakazakii* isolates from different plant and environmental commodities. *Curr. Microbiol.* 74 560–565. 10.1007/s00284-017-1219-9 28258294

[B38] Stojiljković I. Trgovčević Z. e Salaj-S̆mic E. (1991). Tn5-rpsL: a new derivative of transposon Tn5 useful in plasmid curing. *Gene* 99 101–104. 10.1016/0378-1119(91)90039-e 1850707

[B39] Stratev D. Odeyemi O. A. (2016). Antimicrobial resistance of *Aeromonas hydrophila* isolated from different food sources: a mini-review. *J. Infect. Public Health* 9 535–544. 10.1016/j.jiph.2015.10.006 26588876

[B40] Tada T. Hishinuma T. Watanabe S. Uchida H. Tohya M. Kuwahara-Arai K. (2019). Molecular characterization of multidrug-resistant *Pseudomonas aeruginosa* isolates in hospitals in Myanmar. *Antimicrob. Agents Chemother.* 63:e02397–18. 30803967 10.1128/AAC.02397-18 PMC6496111

[B41] Tang Q. Lu T. Liu S.-J. (2018). Engineering the bacterium *Comamonas testosteroni* CNB-1: plasmid curing and genetic manipulation. *Biochem. Eng. J.* 133 74–82. 10.1016/j.bej.2018.01.030

[B42] Tu Q. Yin J. Fu J. Herrmann J. Li Y. Yin Y. (2016). Room temperature electrocompetent bacterial cells improve DNA transformation and recombineering efficiency. *Sci. Rep.* 6:24648. 27095488 10.1038/srep24648 PMC4837392

[B43] Wang P. He D. Li B. Guo Y. Wang W. Luo X. (2019). Eliminating mcr-1-harbouring plasmids in clinical isolates using the CRISPR/Cas9 system. *J. Antimicrob. Chemother.* 74 2559–2565. 10.1093/jac/dkz246 31203365

[B44] Weber T. Wefers B. Wurst W. Sander S. Rajewsky K. Kühn R. (2015). Increasing the efficiency of homology-directed repair for CRISPR-Cas9-induced precise gene editing in mammalian cells. *Nat. Biotechnol.* 33 543–548. 10.1038/nbt.3198 25803306

[B45] Yamamoto S. Sakai A. Agustina V. Moriguchi K. Suzuki K. (2018). Effective removal of a range of Ti/Ri plasmids using a pBBR1-type vector having a repABC operon and a lux reporter system. *Appl. Microbiol. Biotechnol.* 102 1823–1836. 10.1007/s00253-017-8721-7 29318333

[B46] Yin C. Xu L. Li Y. Liu Z. Gu D. Li Q. (2018). Construction of pSPI12-cured *Salmonella enterica* serovar pullorum and identification of IpaJ as an immune response modulator. *Avian Pathol.* 47 410–417. 10.1080/03079457.2018.1471195 29712441

[B47] Yuan Q. Li Y. Xiu L. Zhang C. Fu Y. Jiang C. (2019). Identification of multidrug-resistant *Neisseria gonorrhoeae* isolates with combined resistance to both ceftriaxone and azithromycin, China, 2017–2018. *Emerg. Microbes Infect.* 8 1546–1549. 10.1080/22221751.2019.1681242 31661379 PMC6830194

[B48] Zeng H. Lei T. He W. Zhang J. Liang B. Li C. (2018). Novel multidrug-resistant *Cronobacter sakazakii* causing meningitis in Neonate, China, 2015. *Emerg. Infect. Dis.* 24 2121–2124. 30334728 10.3201/eid2411.180718 PMC6199977

[B49] Zeng H. Li C. Ling N. Zhang J. Chen M. Lei T. (2020). Prevalence, genetic analysis and CRISPR typing of *Cronobacter* spp. isolated from meat and meat products in China. *Int. J. Food Microbiol.* 321:108549. 10.1016/j.ijfoodmicro.2020.108549 32062304

[B50] Zhang T. Zhang X.-X. Ye L. (2011). Plasmid metagenome reveals high levels of antibiotic resistance genes and mobile genetic elements in activated sludge. *PloS One* 6:e26041. 10.1371/journal.pone.0026041 22016806 PMC3189950

